# Correlation Between D-Dimer Concentrations and Thromboelastography in Dogs With Critical Illness: A Retrospective, Cross-Sectional Study

**DOI:** 10.3389/fvets.2022.844022

**Published:** 2022-04-14

**Authors:** Hyun-Jung Han, Jung-Hyun Kim

**Affiliations:** ^1^Department of Veterinary Emergency and Critical Care, Konkuk Veterinary Medical Teaching Hospital, Konkuk University, Seoul, South Korea; ^2^KU Center for Animal Blood Medical Science, Konkuk University, Seoul, South Korea; ^3^Department of Veterinary Internal Medicine, College of Veterinary Medicine, Konkuk University, Seoul, South Korea

**Keywords:** coagulation index, D-dimer, hypercoagulability, inflammation, thromboelastography, tumor

## Abstract

A hospital-based, retrospective, cross-sectional study was performed to assess the correlation of kaolin-activated thromboelastography (TEG) with D-dimer concentrations in 59 dogs with critical illness. Dogs were included if they had one or more serious disease detected upon performing TEG and D-dimer concentration determination based on the same blood sample. According to the coagulation index (CI), the 59 dogs were divided into two groups: a hypercoagulable state, with CI > 4 (44 dogs) and a normocoagulable state, with CI < 4 (15 dogs). Moreover, the 44 dogs with CI > 4 were divided into three sub-groups according to the disease etiology, i.e., inflammatory/tumor (I/T: 25 dogs), hemodynamic compromise (H: 11 dogs), and both conditions (I/TH: 8 dogs). The median values (interquartile ranges) of the CI and D-dimer concentration were 5.46 (4.55–6.33) and 410.25 (74.63–2206.12) in the 44 dogs with CI > 4 and 3.29 (2.92–3.75) and 1164.7 (50–2250.05) in the 15 dogs with CI < 4, respectively. The D-dimer concentration was significantly increased over the reference value in the 44 dogs with a CI > 4, particularly in dogs in the I/T group. It was also significantly increased in the 15 dogs with a CI < 4. D-dimer concentrations did not correlate with any of the TEG parameters in any of the dogs. Given this lack of correlation, the D-dimer concentration may be limited as an indicator of a possible hypercoagulable state in dogs with critical illness.

## Introduction

A hypercoagulable state is caused by the imbalance between the procoagulant and anticoagulant factors which prevents excessive formation of thrombi and initiate fibrinolysis (1–3). In critically ill patients, an imbalance causing a hypercoagulable state frequently occurs due to various critical pathological conditions, such as blood stasis, endothelial injury, inappropriate activation of platelets and soluble clotting factors, and systemic inflammation or endotoxemia, which stimulate the excessive formation of pathologic macro- and microvascular thromboses (3–5). Since such pathological thrombosis could impair oxygen delivery to the cells and tissues, resulting in dysfunction of the affected organ (4) and contributing to an increased risk of mortality, a hypercoagulable state should be identified early to facilitate treatment and prevent thrombosis-associated complications.

Among the various diagnostic tools for coagulation function, viscoelastic testing, such as thromboelastography (TEG) and thromboelastometry, is considered the best tool for the identification of a hypercoagulable state. Although viscoelastic testing is increasingly available and popular in veterinary critical care settings (6), conventional coagulation parameters, such as prothrombin time, activated partial thromboplastin time, and levels of fibrinogen, fibrin degradation product, and D-dimer, are still being used to predict hypercoagulation and thrombosis in veterinary practices because of the simplicity and economic feasibility in determining them. In particular, determination of the level of D-dimer, which is the degradation product of cross-linked fibrin monomers, is the point-of-care test most commonly used in veterinary practices for inferring active coagulation and fibrinolysis (7–9). However, the association of high D-dimer concentrations and thrombosis due to a hypercoagulable state is questionable.

This study investigated the correlation of TEG with D-dimer concentration in dogs with critical illness. The objective of this study was to identify the concentration of D-dimer in critically ill dogs, evaluate its correlation with hypercoagulability as determined by TEG, and finally assess the feasibility of using D-dimer concentration as a diagnostic tool for a hypercoagulable state in dogs with critical illness.

## Materials and Methods

Medical records from the Department of Veterinary Internal Medicine, Konkuk Veterinary Medical Teaching Hospital, of a 15-month period (from April, 2020, to June, 2021) were reviewed retrospectively to identify critically ill dogs for which TEG outcomes and D-dimer concentrations were available. Dogs were included if they were diagnosed with one or more serious diseases and had TEG and D-dimer results. They were excluded from the study if the TEG and D-dimer tests were not performed using the same blood sample obtained during the same visit.

Medical information, such as breed, age, sex, diagnosis, D-dimer concentrations, and outcomes of the TEG parameters was arranged in an electronic spreadsheet database (Supplementary Table 1). The patients were divided into two groups according to their coagulation index (CI) levels, with a hypercoagulable state indicated by CI > 4 and a normo/hypocoagulable state indicated by CI < 4. Moreover, dogs with CI > 4 were divided into three sub-groups according to their pathophysiology, including inflammatory/tumor (I/T), hemodynamic compromise (H), and both these conditions (I/TH). D-dimer concentration was measured using a fluorescent immunoassay using a sodium citrate plasma (Vet chroma^TM^, ANIVET Diagnostic Inc., Chuncheon, Kangwon, South Korea). TEG was performed using a computerized TEG system (TEG®5000, Haemonetics Corporation, Baintree, MA, USA) according to the manufacturer's instructions. The patient's blood samples were tested using citrated kaolin, within 30 min of blood collection. Five parameters were automatically recorded from TEG, including reaction time (R), clot formation time (K), angle, maximum amplitude (MA), and fibrinolysis at 30 min (LY30). The CI was calculated from the equation reported in a previous study: CI = 0.1227(R) + 0.0092(K) + 0.1655(MA) – 0.0241(angle) – 5.0220 (10).

Other coagulation parameters, including the platelet count, prothrombin time, activated partial thromboplastin time, and level of fibrin degradation product, were recorded when available.

For statistical analysis, the one-sample *t* test was used to determine whether the D-dimer concentration was significantly increased over the reference value in dogs. The influence of hypercoagulability on D-dimer concentration was assessed using a Mann–Whitney U test to compare dogs with CI > 4 and CI < 4. Kruskal–Wallis tests with Dunn's multiple comparisons tests were applied to investigate the statistical significance of differences in D-dimer concentrations between dogs with CI < 4 and dogs in the three sub-groups. Furthermore, the correlation between the TEG parameters and D-dimer concentrations was assessed by Spearman's correlation. A logarithmic transformation was performed to obtain a normal distribution for the variables. All the statistical analyses were performed using a commercially available software program (SPSS Advanced Statistics, version 21.0, IBM Germany GmbH, Ehningen, Germany). A *p*-value < 0.05 was considered statistically significant.

## Results

Fifty-nine dogs, including 44 dogs that had a CI > 4 and 15 dogs that had a CI < 4, calculated based on TEG parameters, were identified. The dogs consisted of 29 castrated males, 21 spayed females, 4 intact males, and 5 females. The median age was 10 years (range, 2–18 years), and the breeds included Maltese (*n* = 17), Toy Poodle (*n* = 7), mixed (*n* = 5), Shitzu (*n* = 5), Pomeranian (*n* = 5), Yorkshire Terrier (*n* = 5), Cocker spaniel (*n* = 3), Pekingese (*n* = 3), and one each of Papillon, Shiba Inu, Boston Terrier, Jindo, French bulldog, Great Pyrenees, Bichon Frise, Shetland Sheepdog, and Welsh Corgi. The dogs presented one or more critical diseases, including carcinoma (adrenal, renal, thyroid, mammary gland, tonsil, hepatocellular, and transitional cell), sarcoma (hemangiosarcoma, osteosarcoma, and liposarcoma), other undiagnosed mass (intracranial mass), hormonal conditions (hyperadrenocorticism, hypoadrenocorticism, and hypothyroidism), inflammatory conditions (arthritis, dermatitis, pancreatitis, peritonitis, cholangiohepatitis, gastroenteritis, cystitis, and pneumonia), other hypercoagulable diseases (immune-mediated hemolytic anemia, arterial thromboembolism, lymphoma, urolithiasis, tracheal collapse with or without bronchial collapse, and renal failure), and cardiovascular problems (myxomatous mitral valve degeneration [American College of Veterinary Internal Medicine stage B/C], partial chorda tendineae rupture, pulmonary arterial hypertension, and caval syndrome with heartworm infection).

According to the pathophysiology groups in the 44 dogs with CI > 4, 25 dogs were included in the I/T group, 11 in the H group, and 8 in the I/TH group.

The median and interquartile range of the D-dimer concentrations, CIs, and the TEG parameters for the dogs in each group are shown in Table 1. The median CI was 5.46 (range, 4.05–7.10) and 3.29 (2.92–3.75), and the median platelet count was 435,000 /μL (range, 143,000–1,442,000 /μL) and 354,000 /μL (range, 263,000–629,000 /μL) in the 44 dogs with CI > 4 and the 15 dogs with CI < 4, respectively. No dog had moderate to severe thrombocytopenia.

**Table 1:** Median values (interquartile ranges) of the D-dimer concentration, coagulation index, and thromboelastographic results in 59 dogs with critical illness, consisting of 44 dogs with CI > 4 and 15 dogs with CI < 4.

**Variables**	**Reference interval**	**CI < 4**	**CI > 4**				
			**IT group**	**H group**	**ITH group**	**Total**
Number		15	25	11	8	44
D-dimer (ng/ml)	50–250	1164.7 (50–2250.05)	738.79 (88.78–2384.63)	178.41 (< 50–1304.63)	166.11 (< 50–2152.545)	410.25 (74.63–2206.12)
CI	−4–4	3.29 (2.92–3.75)	5.70 (4.7–6.55)	4.79 (4.35–5.7)	5.61 (4.75–6.18)	5.46 (4.55–6.33)
R (minutes)	1.8–8.6 (6) 1.7–6.1 (7) 1–6 (8)	2.1 (1.3–3)	2.1 (1.75–3.65)	2.5 (1.5–3.3)	1.95 (1.3–2.8)	2.2 (1.6–3.25)
K (minutes)	1.3–5.7 (6) 1.0–3.4 (7) 1–3 (8)	1.5 (1–1.9)	1 (0.8–1.25)	1.1 (0.9–1.6)	0.8 (0.8–0.95)	0.9 (0.8–1.15)
Angle (degrees)	36.9–74.6 (6) 48.5–75.2 (7) 54–74 (8)	71.4 (64.6–76.6)	76.9 (73–79.55)	74.1 (67–76.4)	78.25 (74.95–80.1)	76 (73.15–79.2)
MA (mm)	42.9–67.9 (6) 46.1–64.2 (7) 40–68 (8)	58.5 (56.9–61.7)	74 (68.55–79.05)	68.7 (65–73.2)	73.7 (68.2–77.45)	72.15 (67.05–77.8)
LY30 (%)	0–2 (7)	0.9 (0–4.4)	0.4 (0–1.8)	0.1 (0–1.3)	0.4 (0.05–0.95)	0.3 (0–1.3)

*IT, inflammatory/tumor; H, hemodynamic compromise; ITH, inflammatory/tumor and hemodynamic compromise; CI, coagulation index; R, reaction time; K, clot formation time; MA, maximum amplitude; LY30, fibrinolysis at 30 min*.

**Figure 1:**
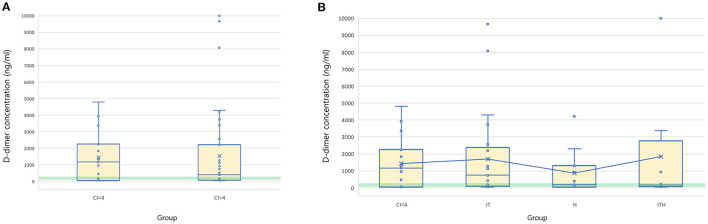
Box and whisker diagrams depicting D-dimer concentration of 44 dogs with CI > 4 and 15 dogs with CI < 4. Median and mean values of D-dimer concentration is compared between the groups of CI > 4 and CI < 4 **(A)**, and CI < 4, IT, H, and ITH **(B)**. The central box represents the values from the lower to upper quartiles and the middle line is the median. The horizontal lines represent minimum to maximum values. X indicate the mean value of the D-dimer concentration in each group. Green semitransparent box represents the normal reference interval of D-dimer concentration (50–250 ng/ml). There is no significant difference of the mean D-dimer concentration between the groups. IT, inflammatory/tumor; H, hemodynamic compromise; ITH, inflammatory/tumor and hemodynamic compromise; CI, coagulation index.

The mean D-dimer concentration was significantly increased over the reference value in both the group with CI > 4 and the group with CI < 4 (Table 2; test value = 250, *p* < 0.05). In the evaluation of sub-groups of the dogs with hypercoagulability (CI > 4), the mean concentration of D-dimer in I/T group was significantly higher than the reference value. The group with CI > 4 included 23 dogs with D-dimer above reference value (15 dogs in I/T group, five dogs in H group, and three dogs in I/TH group), 11 dogs with D-dimer within the reference value (6 dogs in I/T group, two dogs in H group, and three dogs in I/TH group), and 10 dogs with D-dimer below the reference value (four dogs in I/T group, four dogs in H group, and two dogs in I/TH group). In the group with CI < 4, 10 dogs represented the higher concentration of D-dimer over the reference range while five dogs represented D-dimer concentration within or below the reference value (one dog within the value, four dogs below the value). As the result of the comparison of the mean concentration of the D-dimer between the groups, the D-dimer concentration did not differ statistically significantly between dogs with CI > 4 and dogs with CI < 4. Similarly, there were no statistically significant differences between dogs with CI < 4 and those in the I/T, H, and I/TH sub-groups (Figure 1). No statistically significant correlations were found between the D-dimer concentration levels and any of the other TEG parameters (Table 3).

**Table 2:** One-sample *t* test for the evaluation of significant difference between the reference and the measured D-dimer concentrations in dogs with critical illness, consisting of 44 dogs with CI > 4 and 15 dogs with CI < 4.

			**Test value** **=** **250**				**95% Confidence interval of the df**	
			**t**	**df**	**Sig (2-tailed)**	**Mean df**	**lower**	**Upper**
D-dimer	CI < 4		3.020	14	0.009	1191.31	345.13	2037.49
	CI > 4	IT group	2.934	24	0.007	1456.26	431.7	2480.83
		H group	1.563	10	0.149	621.07	−264.39	1506.53
		ITH group	1.301	7	0.235	1601.53	−1310.14	4513.21
		Total	3.460	43	0.001	1273.88	531.3	2016.45

*IT, inflammatory/tumor; H, hemodynamic compromise; ITH, inflammatory/tumor and hemodynamic compromise; df, difference; Sig, Significance; CI, coagulation index*.

**Table 3:** Spearman's correlation coefficients (*p*-values) for D-dimer concentrations and thromboelastography (TEG) variables with coagulation index.

**Variables**			**CI**	**R**	**K**	**Angle**	**MA**	**LY 30**
D-dimers	CI < 4		−0.1261 (0.6518)	0.1309 (0.6383)	0.2974 (0.2784)	−0.2919 (0.2884)	−0.1766 (0.5256)	−0.2621 (0.3407)
	CI > 4	IT group	0.3033 (0.141)	0.0323 (0.879)	0.04887 (0.817)	0.05628 (0.789)	0.2947 (0.153)	−0.09235 (0.661)
		H group	−0.0884 (0.796)	−0.4792 (0.137)	0.1203 (0.720)	0.04187 (0.905)	−0.09305 (0.786)	−0.2537 (0.445)
		ITH group	−0.503 (0.209)	−0.1677 (0.693)	−0.04149 (0.905)	−0.07273 (0.864)	−0.6108 (0.118)	0.5758 (0.141)
		Total	0.06741 (0.664)	−0.1511 (0.328)	0.02198 (0.887)	0.1067 (0.491)	0.0771 (0.619)	0.004143 (0.979)

*IT, inflammatory/tumor; H, hemodynamic compromise; ITH, inflammatory/tumor and hemodynamic compromise; CI, coagulation index; R, reaction time; K, clot formation time; MA, maximum amplitude; LY30, fibrinolysis at 30 min*.

## Discussion

Evaluating hypercoagulability is one of the most important indications for TEG (10). TEG assesses the process of coagulation globally, including its various components, such as plasma, tissue, and phospholipid-bearing blood cell factors, and also provides analysis of fibrinolysis and platelet function (6). Thus, it can yields evidence for the identification of the hypercoagulable state by predicting thrombosis based on prethrombotic changes. In humans, various contexts, such as the use of hormonal contraceptive agents, obesity, post-operative periods, strenuous exercise, uremia, trauma, and cancer, can induce hypercoagulability as identified by TEG (10). In the veterinary field, TEG provides evidence for hypercoagulability in the early stage of disseminated intravascular coagulation (10), parvoviral enteritis (11) protein-losing enteropathy (12) and nephropathy (13), immune-mediated hemolytic anemia (14–16), extrahepatic biliary tract obstruction (17), and neoplasia (18), among other conditions (19). In this type of hypercoagulable state, commonly used TEG variables present typical changes, including a shortened R and K, as well as an increased angle and MA (4, 10, 20).

The CI calculated from the 4 TEG variables, i.e., R, K, angle, and MA, is considered to be a reliable indicator for confirming hypercoagulability in both humans and animals. Previous reports describing the CI in humans have suggested that values above +3.0 reflect a hypercoagulable state (21–24). However, in veterinary studies, a CI above +4.0 is defined as indicating hypercoagulability (10, 13, 14, 25) in various conditions, including immune-mediated hemolytic anemia, extrahepatic biliary tract obstruction, use of nonsteroidal anti-inflammatory drugs, and protein-losing nephropathy in dogs (13, 14, 17, 25).

According to published reference values (10, 13, 14, 25), dogs in this study with a CI > 4 were defined as being in a hypercoagulable state, while dogs with a CI < 4 were considered to be in a normocoagulable state. In the 44 dogs with CI > 4, the I/T group accounted for the largest number of dogs (25 dogs) and showed the highest CI (median 5.7). This is because inflammation and tumors affect all components of Virchow's triad, which promote a hypercoagulable state, as revealed in previous studies (26, 27). During the progression of inflammation and tumors, procoagulant factors, such as coagulation cascade proteins, tissue factors, platelets, and leukocytes, are activated by the vascular damage or abnormal tumor vascularity. This results in increased levels of C-reactive protein, IL-6, IL-8, and TNF-alpha, which stimulate platelet activation, consequently enhancing the prothrombotic state (26, 27). Additionally, in the case of tumors, venous stasis induced by tumor compression could play an important role in inducing hypercoagulability (27). Consequently, dogs in the I/T group in our study exhibited a higher CI than did dogs in the H group, which mostly had hypercoagulability induced by blood stasis caused by myxomatous mitral valve degeneration. We expected that the dogs in the I/TH group might present the highest CI, given the multiple conditions enhancing the hypercoagulable status in this group; however, the median CI of the I/TH group tended to be lower than that of the I/T group. Since the number of dogs included in the I/TH group was relatively small, further studies with a larger number of dogs are needed for a more accurate evaluation of CI in this group.

The association between the increase in the D-dimer concentration and hypercoagulability has long been debated. Several studies have reported the significance of D-dimer in detecting thrombosis. In human medicine, the normal concentration of D-dimer is considered to be a reliable negative predictor allowing the exclusion of a thromboembolic disease (28–31). In dogs, high concentrations of D-dimer are identified in various conditions, including after an operation, internal hemorrhage, liver disease, neoplasia (32, 33), extrahepatic biliary obstruction (32), disseminated intravascular coagulation (34), and other thromboembolic diseases with necropsy evidence of thrombosis (32, 35). However, it remains controversial whether an increased concentration of D-dimer is directly indicative of a hypercoagulable state and thrombosis. D-dimer testing is focused on the detection of increased fibrinolysis rather than on clot formation. In addition to increased clot formation, other factors can increase D-dimer concentrations, such as enhanced fibrinolysis and impaired clearance. In dogs with liver disease, D-dimer concentrations are high due to impaired hepatic clearance of the D-dimer and enhanced fibrinolysis induced by impaired hepatic synthesis of fibrinolysis inhibitors and delayed hepatic clearance of tissue plasminogen activator (36), rather than being secondary to increased fibrin formation (17). Additionally, in human medicine, it has been reported that other factors, such as red blood cell autoagglutination, hypoalbuminemia, and increased corticosteroid levels (of either exogenous or endogenous origin) can also increase the D-dimer concentration (37). Thus, the applicability of D-dimer is limited in related diseases, including protein-losing nephropathy, immune-mediated hemolytic anemia, and hyperadrenocorticism (10). Hence, the specificity of D-dimer is considered to be poor, and the usefulness of an elevated D-dimer concentration for confirming hypercoagulability for a diagnosis of thrombosis is limited (38, 39).

In the present study, dogs with hypercoagulability (CI > 4) presented D-dimer concentrations that were significantly higher than the reference range. Among dogs with CI > 4, those in the I/T group showed significantly higher D-dimer concentrations. Inflammation and tumor-induced vascular damage, abnormal vascularity, and blood stasis could have activated intravascular microthrombus formation and secondary fibrinolysis, which may explain the significant increase in the D-dimer concentration in the dogs in the present study, particularly in the I/T group. Considering the factors, other than thrombosis, that could induce increased D-dimer concentrations, abnormally enhanced fibrinolysis could be excluded, since there was no evidence of hyperfibrinolysis in any of the dogs, as LY30 was in the normal range on TEG. However, several study reported that the TEG alone may not provide sufficient sensitivity for evaluation fibrinolysis, thus tissue plasminogen activator-modified-TEG may be necessary to detect the hyperfibrinolytic potential more accurately (40). The effects of other factors increasing the D-dimer concentration were not strongly considered and the actual increase in the clot formation was only estimated, since hypercoagulability had already been confirmed by the shortened R and K and the angle and MA that exceeded the reference values on TEG.

Significantly higher D-dimer concentrations were also identified in the 15 dogs with CI < 4, which were not regarded as being in a hypercoagulable state based on TEG findings. These dogs were also critically ill, presenting one or more serious conditions, including a tumor, systemic or organ inflammation, and other hormonal-related conditions. Although they did not show overall hypercoagulability on TEG, 10 of these 15 dogs presented markedly increased D-dimer concentrations. We hypothesized that other factors that could increase D-dimer concentrations, rather than hypercoagulability, were affecting these dogs. Six of the 15 dogs were considered to have hyperfibrinolysis, due to the increase in LY30 over the reference values. Most of the other dogs had liver disease, which could enhance fibrinolysis by impairing hepatic synthesis of fibrinolytic inhibitors and reducing the hepatic clearance of D-dimer (36); adrenal problems, such as hyperadrenocorticism and adrenal tumors; and renal diseases, which could induce hypoalbuminemia. These problems have been reported to lead to a marked increase in D-dimer concentrations and are not caused by hypercoagulability. Critically ill dogs often have one or more serious disease that can increase the D-dimer concentration, in addition to increased clot formation. Thus, the usefulness of the D-dimer concentration for evaluating hypercoagulability in critically ill dogs is considered to be limited.

In the present study, the correlation between each TEG parameter and the increase in the D-dimer concentration was investigated to determine the stage of coagulation that is most related to the increase in the D-dimer concentration in dogs with critical illness. However, there was no significant correlation between any TEG parameter and the D-dimer concentration. Most of the previous studies reported similar outcomes of a lack of correlation between traditional coagulation assays and TEG parameters, even though some studies reported inconsistent positive correlations between fibrinogen concentrations and MA, or R and prothrombin time or activated partial thromboplastin time (41, 42). This study could confirm a similar tendency, that the increase in the D-dimer concentration was not consistent with the overall hypercoagulability represented by CI, and it was not proportional to the CI value.

This retrospective study was limited in that dogs with major bleeding disorders were lacking. Since bleeding is a major cause of a marked increase in the D-dimer concentration and induces distinct changes in TEG in both humans and dogs (32, 33, 43), specific correlations between the D-dimer concentration and TEG parameters might be expected. However, none of the dogs identified in our study had noteworthy internal or external bleeding, because the case group used in this study comprised dogs who were brought to the veterinary medical teaching hospital via the department of internal medicine. Since most of veterinary patients with major bleeding disorders are brought to the veterinary emergency center in the hospital first, the bleeding is controlled and stabilized by the department of veterinary emergency and critical care before the patient is taken to other clinical departments. Given the increased clot formation and enhanced fibrinolysis associated with bleeding, which is strongly related to the D-dimer concentration and LY30/60 in TEG, the lack of dogs with bleeding in this study may have resulted in a type 2 error, in that no correlation was observed when one may actually have existed. Therefore, further studies are warranted to identify the correlation between the D-dimer concentration and TEG variables in dogs with major bleeding. Additionally, studies with a larger number of dogs and other traditional coagulation assays, such as those for fibrinogen concentration and platelets, which are known to be related to TEG parameters, are needed to investigate the correlation of changes in TEG parameters in dogs with critical illness.

In conclusion, in dogs with critical illness, the D-dimer concentration did not correlate with the CI or with any parameter on TEG. The D-dimer concentration increased not only in dogs with a hypercoagulable state with a CI > 4, but also in those with a normocoagulable state with a CI < 4. There was no proportional tendency in any of the TEG parameters. Therefore, the D-dimer concentration is limited in its use for estimating the overall hypercoagulable state in critically ill dogs.

## Data Availability Statement

The original contributions presented in the study are included in the article/Supplementary Material, further inquiries can be directed to the corresponding author.

## Ethics Statement

A formal ethical review and approval was not needed because all dogs were sampled for diagnostic and therapeutic purposes with an informed consent of the owner during veterinary visits. Written informed consent was obtained from the owners for the participation of their animals in this study.

## Author Contributions

H-JH was a major contributor to the conceptualization, analysis, interpretation of the data, and manuscript writing. J-HK supervised the study, provided the research planning, and reviewed and edited the manuscript. All those designated as authors contributed substantially to the study design, preparation, and final approval of the manuscript.

## Conflict of Interest

The authors declare that the research was conducted in the absence of any commercial or financial relationships that could be construed as a potential conflict of interest.

## Publisher's Note

All claims expressed in this article are solely those of the authors and do not necessarily represent those of their affiliated organizations, or those of the publisher, the editors and the reviewers. Any product that may be evaluated in this article, or claim that may be made by its manufacturer, is not guaranteed or endorsed by the publisher.
